# Mesenchymal stromal cells in polytrauma: from immune dysregulation to therapeutic potential

**DOI:** 10.3389/fbioe.2026.1892227

**Published:** 2026-10-06

**Authors:** Charles S. Black, Milen Kumar, Renato Miguel Reyes, J. Kent Leach, Mark A. Lee, Håvard Jostein Haugen, Thaqif El Khassawna, Maryam Rahmati, Augustine Mark Saiz

**Affiliations:** 1 Department of Orthopedic Surgery, UC Davis Health, Sacramento, CA, United States; 2 Department of Biomedical Engineering, University of California, Davis, Davis, CA, United States; 3 Department of Biomaterials, Institute of Clinical Dentistry, University of Oslo, Oslo, Norway; 4 Experimental Trauma Surgery, Justus-Liebig University Giessen, Giessen, Germany; 5 School of Pharmacy, The University of Jordan, Amman, Jordan

**Keywords:** bone regeneration, fracture, inflammation, mesenchymal stromal cells, polytrauma

## Abstract

Polytrauma, defined as life-threatening injuries to multiple organ systems with an Injury Severity Score greater than 16, presents significant challenges for fracture healing, with nonunion rates substantially higher than in isolated fractures. The systemic inflammatory response syndrome (SIRS) and subsequent immune dysregulation characteristic of polytrauma create a hostile microenvironment that impairs normal bone regeneration. Mesenchymal stromal cells (MSCs; also termed mesenchymal stem/stromal cells) play critical roles in fracture healing through immunomodulation, osteogenic differentiation, and promotion of angiogenesis. However, emerging evidence suggests that MSC-mediated processes may be compromised in polytrauma settings. Importantly, direct evidence for endogenous MSC dysfunction in human polytrauma remains limited: much of the current understanding is inferred from isolated-fracture models, *in vitro* inflammatory-stimulation studies, and animal polytrauma systems rather than from controlled human trauma data. This review examines the mechanisms by which polytrauma is associated with disruption of MSC function, including altered macrophage polarization, excessive neutrophil activation, reduced regulatory T cell populations, and complement-mediated MSC injury. We synthesize the current understanding of MSC immunomodulatory effects in normal fracture healing and contrast this with their dysfunction in polytrauma, where MSCs adopt pro-inflammatory phenotypes and demonstrate impaired homing. Clinical applications of MSC-based therapies are discussed, including bone marrow aspirate concentrates and tissue-engineered constructs, alongside their potential for systemic immunomodulation in trauma patients. Throughout, we distinguish endogenous MSC behavior in polytrauma from exogenous MSC-based therapies, and we separate various MSC-based therapies as biologically and translationally distinct concepts. Finally, we outline critical knowledge gaps and propose future research directions, including development of representative polytrauma models, optimization of MSC delivery methods, and exploration of MSC-derived extracellular vesicles as therapeutic alternatives to improve outcomes in polytraumatized patients.

## 1 Introduction

Traumatic injuries accounted for nearly 7% of all deaths in the United States in 2022 (Xu et al., 2025). Of these, polytrauma has a particularly high mortality rate. Polytrauma is defined as potentially life-threatening injuries to multiple organ systems from a single event and an Injury Severity Score (ISS) of >16 (Champion et al., 1990). Among the most common injuries in polytrauma are extremity fractures, with almost 60% of polytraumatized patients being diagnosed with one or more extremity fractures (Banerjee et al., 2013). Improper or complete lack of fracture healing, termed malunion and nonunion, is more common in polytrauma patients than patients with an isolated fracture (Bastian et al., 2016), often requiring additional surgeries and greater patient burden (Hoff et al., 2017). The likely cause of poor fracture healing and patient outcomes in polytrauma patients is that the immune response is not the sum of all the injuries, but rather an imbalance between the pro- and anti-inflammatory responses. Polytrauma incurs an initial systemic inflammatory response syndrome (SIRS), characterized by widespread endothelial injury and release of damage-associated molecular patterns (DAMPs) (Keel and Trentz, 2005). These factors trigger cytokine storms, impair angiogenesis, and obstruct tissue regeneration, causing immune dysregulation both locally and systemically. This initial response is followed by a sustained period of immune tolerance, making it difficult for the body to resolve the issues caused by the initial inflammatory response (Keel and Trentz, 2005). Significant complications can arise if this imbalance is left untreated, leading to multi-organ dysfunction syndrome (MODS) and multi-organ failure (MOF) (Keel and Trentz, 2005). Successful discovery and application of immune-regulating therapies could help to improve outcomes of polytraumatized patients in the future.

Mesenchymal stromal cells (MSCs) are a type of multipotent progenitor cell that play a role in a variety of different processes in the body. MSCs are in the highest concentration in the bone marrow but can be found in many other tissue compartments, such as the umbilical cord, perivascular regions of connective tissue, muscle and adipose tissue and more (Hass et al., 2011). Though the resident site can influence MSC actions, their influence is generally anti-inflammatory. They primarily act through direct cell-to-cell contact, secretion of extracellular vesicles (EVs) and cytokines and differentiation into a variety of cell lineages (Hoff et al., 2017). However, MSCs have also demonstrated the ability to secrete pro-inflammatory cytokines in certain environments (Raicevic et al., 2010; Saiz et al., 2025a), illustrating their context-dependent nature. The roles they play in immunomodulation and fracture healing have been studied extensively: an important review by Ma et al. (2014) synthesized the known immunomodulatory roles that MSCs have in the settings of various inflammatory diseases (Ma et al., 2014). The paper highlights the influence of indoleamine 2,3-dioxygenase (IDO) on MSCs, the timeline of MSCs in the bloodstream and bidirectional interactions between MSCs and the rest of the immune system (Ma et al., 2014). In the field of regenerative medicine, a meta-analysis of studies using MSCs as an adjunct therapy in patients with fracture nonunion found that MSC application was associated with improved healing time compared with bone graft at 3 and 6 months (Cui et al., 2025). Further improvements were seen when MSCs were applied with a scaffold (Ma et al., 2014). Additionally, Medhat et al. (2019) reviewed the different roles that MSCs have in isolated fracture healing, highlighting their specific effects on various immune cells and the mechanisms by which they exert these effects (Medhat et al., 2019). These functions, along with their demonstrated pro-angiogenic capabilities (Arthur and Gronthos, 2020) make them a good candidate for investigation in the context of polytrauma. However, the effects of native MSCs in polytrauma remain relatively uncharacterized. Given the differences in the immune response between polytrauma and isolated fractures (Saiz et al., 2025b), it is unreliable to extrapolate from one setting to the other. In this review, we provide an overview of the role of MSCs in fracture healing, review the literature about the immune response in polytrauma and how MSCs might play a role, outline the clinical applications of MSCs in polytrauma conditions so far, and give recommendations on how the field should progress in the future.

To keep these distinctions clear; this review consistently separates six biologically and translationally distinct concepts: (1) the behavior of endogenous MSCs within the polytrauma environment; (2) exogenous MSC therapy administered after polytrauma; (3) MSC-rich bone marrow aspirate concentrate (BMAC); (4) culture-expanded MSC products; (5) MSC-derived extracellular vesicles (MSC-EVs); and (6) biomaterial/scaffold-assisted MSC delivery. For each mechanism and application, we state what is directly established, what is inferred, and what remains unknown, and we indicate whether supporting data derives from human polytrauma, human fracture/nonunion, preclinical polytrauma models, *in vitro* MSC stimulation, or extrapolation from other inflammatory conditions such as ARDS and sepsis.

## 2 Literature review methodology

This narrative review surveyed the peer-reviewed literature on MSC immunomodulation in polytrauma and fracture healing from database inception through 2 November 2025 (final search date), with emphasis placed on the most recent decade where available. Seminal earlier studies were included when necessary to establish foundational concepts (e.g., phases of fracture healing, core osteoimmune mechanisms) and to contextualize newer mechanistic and translational findings.

To identify relevant studies, keyword combinations were used in Embase, PubMed, and Web of Science. Search terms included, but were not limited to: “polytrauma”, “multiple trauma”, “systemic inflammation”, “immune dysregulation”, “SIRS”, “CARS”, “fracture healing”, “fracture repair”, “nonunion”, “delayed union”, “mesenchymal stem cells”, “mesenchymal stromal cells”, “immunomodulation”, “macrophage polarization”, “M1”, “M2”, “neutrophil”, “NETs/NETosis”, “T cells”, “regulatory T cells”, “cytokines”, “TNF-α”, “IL-6”, “complement”, “homing”, “CXCR4”, and “extracellular vesicles/exosomes”. Reference lists of included articles were additionally screened to capture relevant studies not retrieved in the primary searches. Boolean strings were not standardized, and PRISMA flow metrics were not recorded due to the need to capture the full scope of an expansive knowledge base.

Studies were included if the full text was published in English and produced original research on fracture healing, the immune response to or clinical implications of polytrauma, the role of MSCs in fracture healing or polytrauma, or clinical use of MSCs in fracture healing. Studies not relevant to these topics were excluded. No studies were excluded based on study type (i.e., *in vitro*, *in vivo*, observational human, and clinical studies were all eligible) or data availability in order to capture the full scope of the relevant literature. Records from the three databases were imported into a reference manager and deduplicated before screening. Review articles meeting eligibility criteria were used to identify primary sources via reference-list screening and as direct sources of contextual and background information.

Titles/abstracts and full texts were independently screened by two reviewers, with disagreements resolved by consensus; a third reviewer adjudicated cases where consensus could not be reached. This dual-reviewer process, with third-reviewer arbitration for disagreement, served as the bias mitigation system. Data were extracted on MSC source and/or MSC-based intervention (including cell-free derivatives when applicable), study type (preclinical vs. clinical), immune-related outcomes, and relevance to polytrauma physiology and/or fracture repair.

Some limitations exist within our review method. As this is a narrative rather than a systematic review, a formal quantitative synthesis (meta-analysis) was not performed, and a formal risk of bias/quality appraisal of individual studies was not conducted. Owing to heterogeneity in models, interventions, and outcomes, and to the deliberate integration of *in vitro*, animal, observational human, and clinical evidence, findings were synthesized qualitatively and organized by dominant immunological mechanism. The exclusion of non-English studies may introduce language bias. Similarly, restricting inclusion to studies with freely available full text may have introduced an additional source of selection bias.

## 3 Mesenchymal stem cells and fracture healing

Fracture healing is a complex biological process involving an orchestrated interplay between inflammation, tissue regeneration, and remodeling to restore structural and functional bone integrity. In the context of fracture healing, MSCs exhibit immunoregulatory, osteogenic and angiogenic functions at the fracture site. Their bidirectional interactions with cytokines, immune cells and other cells present at the fracture site demonstrate a context-dependent nature.

### 3.1 Fracture healing overview

Fracture healing occurs in five progressive and overlapping stages, the first of which is hematoma formation. This prevents further hemorrhage and initiates the immune response through localized activation of inflammatory cells while creating a provisional matrix (Loi et al., 2016). The second stage involves acute inflammation, during which neutrophils and macrophages of a pro-inflammatory phenotype (M1) infiltrate the site to clear debris and form a vascularized connective tissue bridge (Loi et al., 2016). Next is soft callus formation; through endochondral ossification, the bridge is gradually transformed into a soft cartilaginous callus, stabilizing the fracture and supporting neovascularization (Zhang et al., 2017; Sheen et al., 2023). This soft callus is then mineralized into a hard callus composed of woven bone. Though structurally weaker than mature bone, the hard callus is crucial for mechanical support and load transfer as the fracture site begins adapting to biomechanical stressors (Maruyama et al., 2020). Finally, bone remodeling consists of gradual replacement of the hard callus with organized lamellar bone, a process mediated by osteoclasts and osteoblasts to reshape the callus according to mechanical demands and original bony architecture (Maruyama et al., 2020). MSCs play a critical role in this process, as the following subsections demonstrate.

### 3.2 Homing and migration to the fracture site

The ability of MSCs to migrate to the fracture site is critical for their influence on the fracture microenvironment. MSC migration begins at the hematoma stage and continues into the bone formation phase, when recruitment begins to decline (Garg et al., 2017). The most potent inducer of MSC migration is stromal cell-derived growth factor 1 (SDF-1), which originates in the periosteum of injured bone and recruits MSCs to the injury site by binding to C-X-C chemokine receptor type 4 (CXCR4) on the surface of MSCs (Kitaori et al., 2009). Additionally, pro-inflammatory cytokines such as tumor necrosis factor-α (TNF-α) and interleukin-1β (IL-1β) from the injury site can stimulate the migration of MSCs (Lin et al., 2017). Lin et al. gives a detailed description of the various homing mechanisms of MSCs (Lin et al., 2017). Migration of MSCs is a critical step in the repair process, as it allows MSCs to exert a powerful local influence on the fracture microenvironment.

### 3.3 Immunomodulation at the fracture site

The immunomodulatory role of MSCs at the fracture site primarily involves facilitating the transition from a pro-inflammatory/catabolic to an anti-inflammatory/anabolic state. Upon arrival at the fracture site, MSCs sense inflammatory cytokines via receptors such as TNFR1/2, IL-1R, and Toll-like receptors (TLR)s. These inputs activate intracellular pathways including NF-κB, PI3K/Akt, and JAK/STAT, enabling MSCs to exert broad paracrine effects. MSCs produce prostaglandin E2 (PGE2), IL-10, transforming growth factor-β (TGF-β), heme oxygenase-1 (HO-1), IDO and upregulate PD-1 receptors on T regulatory cells (Tregs) to suppress excessive immune activation. Notably, these factors encourage macrophage polarization toward the anti-inflammatory M2 phenotype, inhibit T cell proliferation and enhance Treg formation and immunosuppressive activity (Xu et al., 2023; Stewart, 2019; Gibon et al., 2017) (Figure 1a). Additionally, MSCs suppress neutrophil activity and migration to the fracture site through expression of TNF-α-stimulated gene/protein 6 (TSG-6) (Medhat et al., 2019; Xu et al., 2023). This suppression is established in isolated fracture and non-trauma models; whether it holds in polytrauma is inferred rather than demonstrated. MSCs also release extracellular vesicles (EVs) that contain immunomodulatory microRNAs, such as miR-21 and miR-146a, which directly repress the expression of inflammatory mediators in target immune cells (Gibon et al., 2017). However, in contrast with TSG-6 expression, MSCs can express IL-8 and macrophage migration inhibitory factor (MIF) to increase neutrophil migration to the fracture site (Xu et al., 2023). Together, these actions demonstrate the context-dependent nature of MSCs and position them as a critical mediator of the immune response to fracture.

**FIGURE 1:**
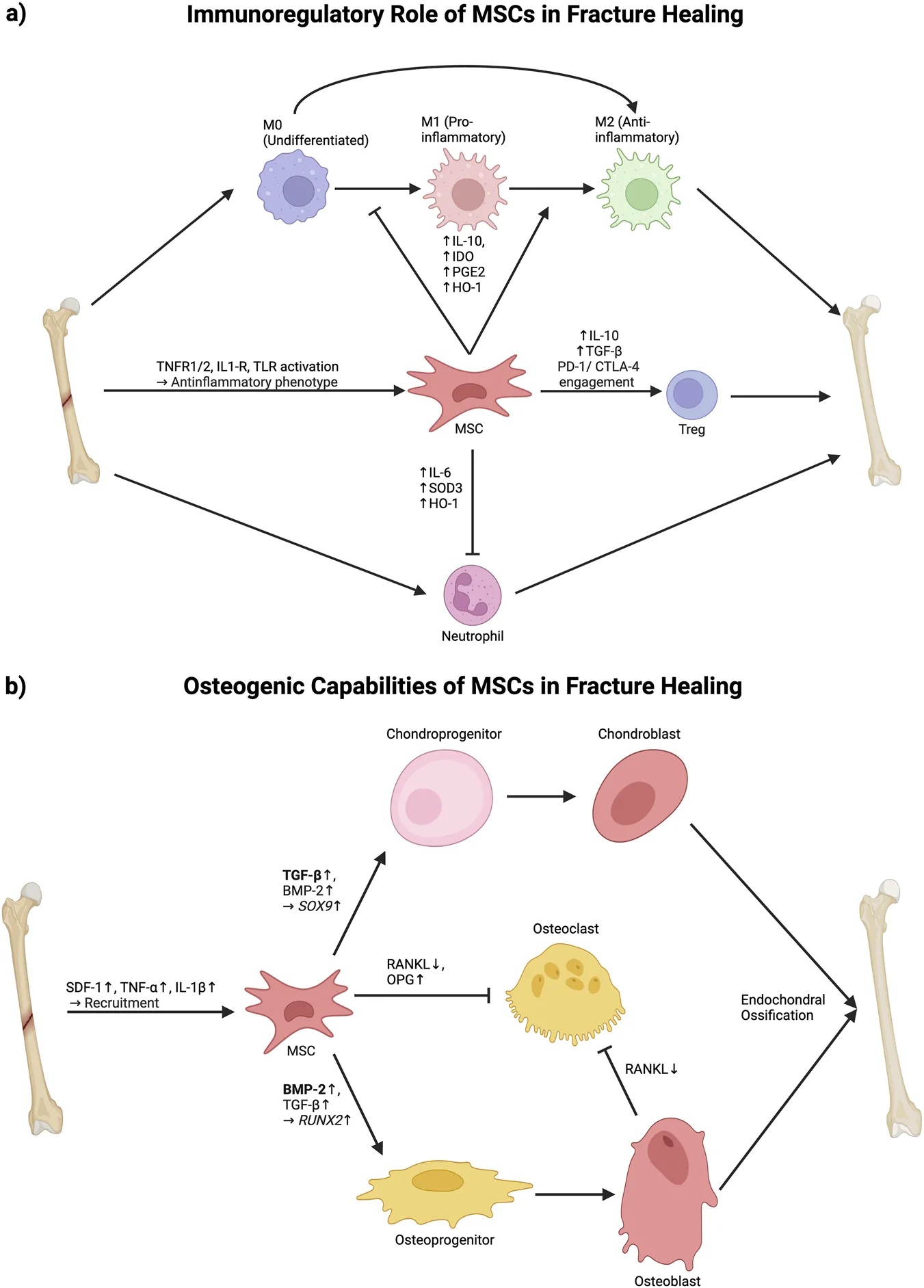
Immunoregulatory and Osteogenic Roles of MSCs in Isolated Fracture Healing. An overview of the potential roles of MSCs in fracture healing. **(a)** MSCs primarily exert an anti-inflammatory influence on the fracture microenvironment, most notably impacting the effects of neutrophils macrophages, and T regulatory cells (Tregs). **(b)** MSCs are a significant source of osteogenic cells in fracture repair, most notably chondroblasts and osteoblasts, which contribute to bone formation and decrease bone resorption. Created using BioRender.

### 3.4 Osteogenic and chondrogenic differentiation

MSCs also have the capacity to directly affect osteogenesis through differentiation into osteoblasts and chondroblasts and suppression of osteoclast activity. At physiological conditions, MSCs can either enhance or suppress osteoclast activity through osteoprotegerin (OPG) or receptor activator of nuclear factor kappa-B ligand (RANKL). When a fracture occurs, MSCs decrease RANKL secretion and increase OPG secretion to inhibit osteoclast formation and activity (Sharaf-Eldin et al., 2016). With regards to the differentiation potential of MSCs, the critical regulators are TGF-β and bone morphogenetic proteins (BMPs), primarily BMP-2 (though other BMPs play a role in osteogenic differentiation of MSCs) (Garg et al., 2017) (Figure 1b). Chondrogenic differentiation primarily relies on *SOX9* upregulation by TGF-β. In contrast, osteogenic differentiation is induced by *RUNX2* via BMP-2, though both factors are involved in each process, as are other BMPs (Peng et al., 2023). Though TGF-β plays a greater role in differentiation into chondroprogenitors and different BMPs are involved in each process, both factors are essential in regulating the differentiation potential of MSCs (Garg et al., 2017; Peng et al., 2023; Scarfì, 2016). A review from Scarfi (2016) includes additional description of how BMPs have been studied in osteogenesis and chondrogenesis, both *in vitro* and *in vivo* (Scarfì, 2016). BMP-2 binds to BMP receptor (BMPR) I and II, activating the Smad1/5/8 pathway and ultimately upregulating *RUNX2*, a key transcription factor for MSC differentiation into osteoblasts (Stewart, 2019; James, 2013; Halloran et al., 2020). Although MSCs are not the only source of osteoblasts and chondroblasts, the absence of TGF-β and BMP-2 prevent MSCs from differentiating and severely impairs fracture healing (Garg et al., 2017).

### 3.5 Support of angiogenesis

MSCs also contribute to the revascularization of the broken bone, allowing osteogenic factors to traffic to the fracture site and contribute to the repair process. Genova et al. (2019) demonstrated that crosstalk between MSCs and endothelial cells (ECs) leads to an increased secretion of pro-angiogenic factors such as vascular endothelial growth factor (VEGF), Angiopoietin (ANG) one and 2, TGF-β and platelet-derived growth factor subunit b (PDGFb) (Genova et al., 2019), thereby increasing EC count. However, EC secretion of BMP promotes differentiation of MSCs, simultaneously limiting their angiogenic capacity (Canali et al., 2017), a further indication of the relevance of crosstalk between MSCs and fracture microenvironment.

## 4 Immune response to polytrauma leading to nonunion

In response to injury, the immune system initiates a tightly orchestrated response that is essential for initiating tissue repair and fracture healing. However, excessive or prolonged activation of both the innate and adaptive immune systems can disrupt this balance, leading to impaired bone regeneration and potentially progressing to fracture nonunion (Figure 2) (Gibon et al., 2017; Eggenhofer et al., 2012; White et al., 2016; Muire et al., 2020).

**FIGURE 2:**
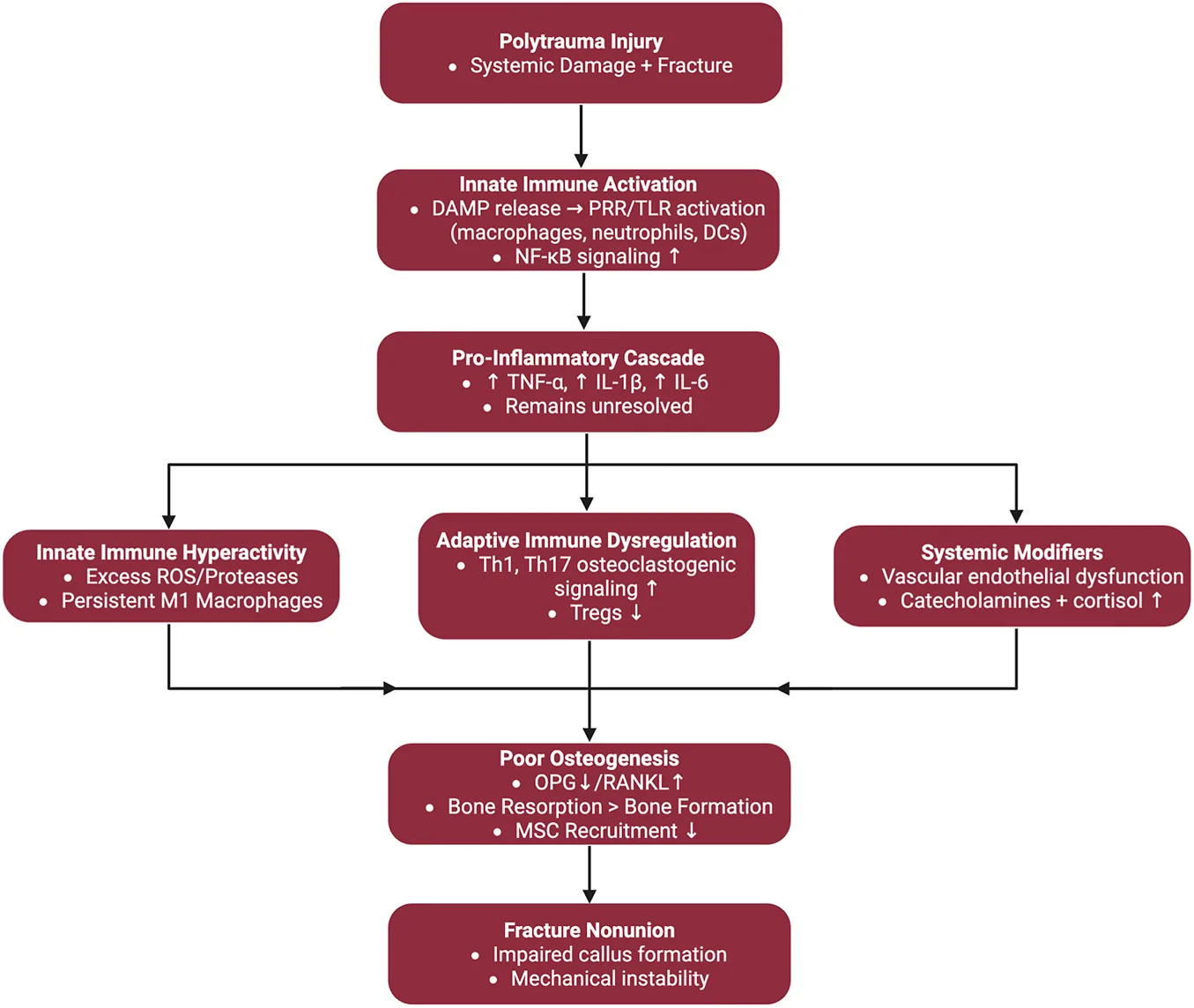
Progression of Polytrauma to Fracture Nonunion. An overview of the primary mechanism by which fracture nonunion occurs due to polytrauma. Atrophic nonunion is most common in polytrauma due to lack of callus formation. Created using BioRender.

### 4.1 Overview of fracture nonunion

Fracture nonunion is a significant complication in orthopedic trauma. In human patients, nonunion can be defined as a fracture that has not healed after 9 months and has not shown any signs of healing for at least three consecutive months (Toosi et al., 2018; Calori et al., 2017). They are most frequently observed in long bones such as the tibia and femur, where the extended healing period is typically beyond 6 months (Toosi et al., 2018). Nonunion is generally classified into two primary types, hypertrophic and atrophic. Hypertrophic nonunion typically results from insufficient mechanical stabilization despite preserved biological activity (Stewart, 2019). Atrophic nonunion results from a biologically inactive healing environment; Even when mechanical stabilization is adequate, the biological response to initiate healing remains impaired, often requiring interventions targeting the vascular and cellular microenvironment (Stewart, 2019; Rupp et al., 2019). Of these two types, atrophic nonunion is more common in polytrauma due to a lack of callus formation (Sardesai et al., 2021).

### 4.2 Innate immune response and the RANKL axis

Immediately after a traumatic injury, damaged and necrotic tissue releases damage associated-molecular patterns (DAMPs), which are recognized by pattern recognition receptors (PRRs), most notably TLRs on resident macrophages and dendritic cells. This interaction triggers a pro-inflammatory signaling cascade driven by NF-κB, leading to the release of cytokines such as TNF-α, IL-1β, and IL-6 (Gibon et al., 2017). These cytokines subsequently enhance RANKL expression and activate the RANK/RANKL/OPG axis, a pivotal pathway in osteoclast differentiation. Sustained RANKL activation is common in polytrauma, promoting excessive osteoclastogenesis and bone resorption, undermining structural integrity and preventing fracture healing (Gibon et al., 2017; Pajarinen et al., 2019).

A hallmark of the polytrauma immune profile is the biphasic systemic response. The initial phase is driven by the rapid release of pro-inflammatory cytokines and is characterized by a surge in innate immune, referred to as SIRS. This triggers a compensatory anti-inflammatory response syndrome (CARS), in which a counter-regulatory mechanism prevents the activation of the adaptive immune system and resolution of the initial inflammatory response. Together, these systemic shifts disrupt the balance between inflammation and resolution both at the fracture site and on a systemic level (Muire et al., 2020; Pajarinen et al., 2019).

In isolated fractures, the innate immune response at the fracture site initiates with hematoma formation. Neutrophils are the first immune cells to infiltrate the hematoma, releasing proteolytic enzymes and reactive oxygen species (ROS). Neutrophils also secrete chemokines, such as CCL2 and CXCL8, which recruit monocytes that later differentiate into pro-inflammatory M1-like macrophages. These M1-like cells maintain their inflammatory phenotype via STAT1, NF-κB, and IRF5 pathways. By secreting pro-inflammatory cytokines and PGE2, M1-like macrophages attract MSCs and enhance their differentiation potential through secretion of oncostatin M (OSM) promoting osteogenesis. Subsequently, a phenotypic transition from M1-like to M2-like macrophages occurs in the later phases of inflammation and is essential for proper healing. M2-like macrophages, promoted by IL-4/IL-13 and STAT6 signaling, secrete IL-10, TGF-β, VEGF, and IGF-1, all of which support angiogenesis, extracellular matrix formation, and osteoblast maturation. In polytrauma, the initial inflammatory response is both heightened and unresolved, resulting in excessive neutrophil and M1-like macrophage activity at the fracture site. This disrupts the fracture healing timeline by prolonging the resorptive phase and preventing the transition to bone formation, increasing the risk of nonunion (Gibon et al., 2017; Pajarinen et al., 2019; Maniar et al., 2025). Specifically, atrophic nonunion is more common in polytrauma due to a lack of callus formation.

### 4.3 Adaptive immune response

The adaptive immune system also plays a key role in fracture healing. CD4^+^ T cells are recruited in the later stages of the inflammatory phase. Among these, Th1 cells secrete IFN-γ and Th17 cells release IL-17A and IL-22. These cytokines further promote osteoclastogenesis via upregulation of RANKL expression. These pathways are controlled by transcription factors like STAT3 and RORγt. In contrast, regulatory T cells (Tregs) exert anti-inflammatory effects via FOXP3, promoting the release of IL-10 and TGF-β. These cytokines are crucial for suppressing inflammation and facilitating tissue regeneration. In polytrauma patients, reduced Treg populations are frequently observed, removing a key regulatory checkpoint and shifting the immune balance toward a destructive, catabolic state (Gibon et al., 2017; Eggenhofer et al., 2012; White et al., 2016; Muire et al., 2020). Additionally, B cell dysregulation contributes to impaired bone healing. Inflammatory conditions can skew the RANKL to OPG secretion ratio, favoring osteoclast activation and preventing the shift to an anabolic state, delaying the healing timeline and increasing the risk of nonunion (Gibon et al., 2017).

### 4.4 Vascular and endocrine contributions

Other systems can also play a role in the development of nonunion. Polytrauma-induced endothelial injury impairs microvascular perfusion, limiting delivery of oxygen, nutrients, immune cells, and MSCs to the fracture site. This ischemic environment hampers angiogenesis and osteoblast proliferation, thereby exacerbating nonunion risk (Stewart, 2019; Muire et al., 2020). Another factor to consider is the endocrine system’s response to trauma, as this also influences immune-bone interactions. Elevated cortisol and catecholamines during stress can suppress T cell activity, inhibit MSC proliferation, and bias MSC differentiation toward adipogenesis rather than osteogenesis (Gibon et al., 2017; Wildemann et al., 2021). These systemic hormonal effects can amplify the detrimental consequences of immune dysregulation in fracture healing.

In summary, polytrauma creates a complex immune environment in which prolonged inflammation, systemic adaptive immune suppression, endothelial dysfunction, and hormonal stress responses all converge to impair fracture healing. If this delicate immune/bone/vascular balance is disrupted, chronic inflammation and nonunion are likely outcomes (Loi et al., 2016; Stewart, 2019; Muire et al., 2020; Thomas and Kehoe, 2023).

## 5 The immunomodulatory effects of MSCs in polytrauma

MSCs play an important role in facilitating the transition from a pro-inflammatory to an anti-inflammatory environment, making them critically important in polytrauma settings. However, there is evidence that mechanisms that are regulated by MSCs during an immune response may not function properly in polytrauma. This suggests that MSC function may be impaired in polytrauma (Figure 3), although a direct investigation into endogenous MSC function in human polytrauma has not been performed and is needed to confirm this inference.

**FIGURE 3:**
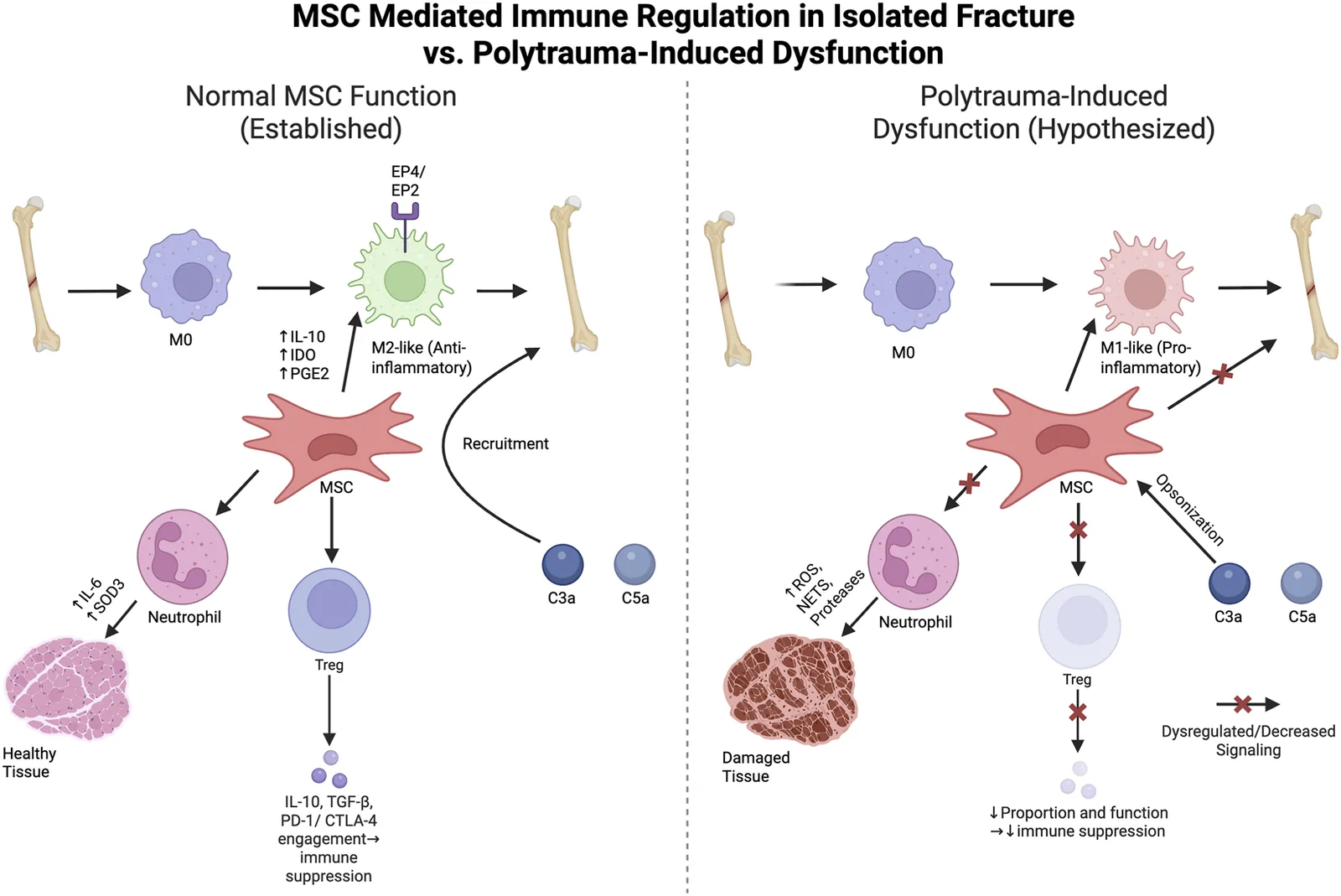
Overview of Dysfunctional MSC-mediated processes. Schematic detailing the contrast between MSC function in the healing process of an isolated fracture (established) and a polytrauma fracture (hypothesized). There are several hypothesized alterations in MSC-mediated processes in polytrauma, most notably a decrease in Treg proportion and function, excess neutrophil activation and apoptosis, and increased pro-inflammatory macrophage concentration. The complement system also shows altered functionality, opsonizing MSCs at a higher rate than normal rather than recruiting them to the fracture site. Created using BioRender.

A critical interpretive caveat applies to this section. The findings below describe a hostile systemic immune environment after polytrauma in which MSC-mediated processes appear insufficient or altered. They do not, however, establish that endogenous MSCs are the primary driver of these immune abnormalities. Where evidence is associated or extrapolated from isolated-fracture or *in vitro* systems, this is stated explicitly, and the evidence tier for each mechanism is summarized in Table 1.

**TABLE 1:** Therapeutic and delivery studies of MSCs and MSC-based products.

Study and model	Product/route	Outcome (brief)	Limitations/translational interpretation	Ref
Hernigou et al., tibial atrophic nonunion (n = 60)	Percutaneous autologous BMAC	∼88% union; progenitor dose correlated with healing	Isolated nonunion, not polytrauma; observational	(Hernigou et al., 2006)
Desai et al., tibial delayed/nonunion (n = 49)	BMAC + DBM or BMP-2	Union in most; DBM > BMP-2 in cohort	Retrospective; isolated nonunion	(Desai et al., 2015)
Orozco Delclós et al., long-bone nonunion (Phase II)	Culture-expanded adipose MSC + TCP; auto vs. allo	83% union; auto ≈ allo; no serious AEs	Controlled but isolated nonunion; small n; not polytrauma	(Orozco Delclós et al., 2024)
Matthay et al., START (Phase 2a)	Allogeneic BM-MSC IV, ARDS	Safe; no mortality benefit in small trial	Extrapolated (ARDS); safety not efficacy	(Matthay et al., 2019)
He et al., severe sepsis (Phase 1)	UC-MSC IV	Safe; reduced circulating cytokines	Extrapolated (sepsis); not efficacy-powered	(He et al., 2018)
Saiz et al., murine polytrauma	MSC systemic vs. local HA hydrogel	Local MSC superior; IV MSC lung-trapped	Preclinical polytrauma; direct but animal-only	(Saiz et al., 2025a)
Saiz et al., murine polytrauma	HA vs. HA + P2 vs. HA + MSC + P2, local	MSC + P2 enhanced mineralization; MSC anti-inflammatory dominant	Preclinical polytrauma; animal-only	(Saiz et al., 2025c)
Valade et al., traumatic hemorrhagic-shock models (review)	MSC-derived EVs (cell-free), systemic	MSC-EVs reduced lung injury and improved outcomes by modulating inflammation	Preclinical only; animal hemorrhagic shock, not polytrauma fracture; EV heterogeneity/hemocompatibility unaddressed	(Valade et al., 2021)

### 5.1 Macrophage polarization

A known mechanism of MSCs is their ability to polarize macrophages from an M1-like to an M2-like phenotype. An important note is that macrophage phenotype exists along a spectrum, with M2-like describing those that have more anti-inflammatory properties and M1-like describing those with more pro-inflammatory properties. These are not permanent or exclusive states, as macrophages can adopt characteristics of both simultaneously (Ji et al., 2025). In a typical inflammatory response to fracture, MSC secretion of PGE2, IDO and IL-10 interact with EP2 and EP4 receptors on macrophages to prevent differentiation of undifferentiated (M0) macrophages to M1-like macrophages, enhance differentiation from M0 to M2 and induce the shift from M1-like to M2-like (Pajarinen et al., 2019). This shift plays a key role in managing the inflammatory cascade and preventing bone hyperresorption. A previous study from our group found that the percentage of macrophages at the fracture site is elevated in polytrauma, though bone healing was still impaired (Saiz et al., 2025b). Whether an impaired transition from M1 to M2 is the key reason behind this is not known, as macrophage phenotype data were not collected. However, it is possible that malfunctioning MSC-macrophage interactions could contribute to impaired bone healing. Though it has been demonstrated that MSC concentration is reduced in the peripheral blood in polytrauma, their presence and role at the fracture site in polytrauma are not well established (Wiegner et al., 2018).

### 5.2 Neutrophil regulation

MSCs also interact with neutrophils, both at the fracture site and systemically. MSCs modulate neutrophil hyperactivity and protect against neutrophil apoptosis. Raffaghello et al. demonstrated that MSCs prevent neutrophil apoptosis and hyperactivation of oxidative metabolism while preserving their effector functions through IL-6 secretion (Raffaghello et al., 2008). Additionally, Jiang et al. found that MSCs prevent neutrophil death, neutrophil extracellular trap (NET) formation and spillage of neutrophil derived proteases through secretion of extracellular superoxide dismutase (SOD3) both *in vivo* and *in vitro* (Jiang et al., 2016). However, neutrophil hyperactivity is common in polytrauma, leading to extended release of cytotoxic and antimicrobial factors, causing cell and tissue injury and death before undergoing apoptosis (Martí‐Chillón et al., 2023). Large scale neutrophil death causes release of cytotoxic products, further damaging surrounding tissue. Normally, MSCs combat this through a similar mechanism to that which occurs in fracture healing, preventing neutrophil leakage and secondary tissue damage. However, these mechanisms appear dysregulated in polytrauma, evidenced by the incidence of secondary tissue injuries from neutrophil leakage and hyperactivity such as acute respiratory distress syndrome (ARDS) and acute kidney injury (AKI) (Martí‐Chillón et al., 2023). These clinical sequelae reflect a hostile neutrophil environment, yet they do not themselves prove that endogenous MSC restraint of neutrophils is the failing step.

### 5.3 Regulatory T cells

Regulatory T cells (Tregs) are critical in maintaining immunologic homeostasis, and their expansion capacity is influenced in part by MSCs. Tregs are generally immunosuppressive, primarily acting as lymphocyte inhibitors through secretion of IL-10 and TGF-β and contact at the PD-1, CTLA-4 and LAG-3 receptors (Yan et al., 2014). MSCs are key regulators of Treg dynamics in fracture healing, both at the fracture site and systemically. To date, there is not a significant amount of literature examining the activity of Tregs at the fracture site in polytrauma. However, Serve et al. analyzed Treg dynamics in the peripheral blood of polytraumatized humans and pigs and found that Treg proportion was decreased in both at 72 h (Serve et al., 2018). A recovery in proportion was made in the porcine and clinical models, though a recovery in absolute cell count was only seen in the human patients and not in the porcine model (Serve et al., 2018). As such, decreased Treg concentration in the peripheral blood is consistent with, but does not by itself, demonstrate impaired MSC-mediated Treg support in polytrauma; reduced Treg numbers may equally reflect the broader hostile immune milieu.

### 5.4 MSC secretome and delivery route

A recent study from our group investigated the secretory profile of MSCs and the effects of local and systemic administration in polytrauma. MSCs exposed to polytrauma serum *in vitro* demonstrated significantly increased secretion of pro-inflammatory cytokines, such as TNF-α, GM-CSF, IFN-γ, IL-6, IL-1α, IL-1β, and chemokines MCP-1, MCP-5 and MIP-1α, compared to those exposed to isolated fracture serum. Notably, at day 7, secretion of IL-17 and IL-12p70 remained elevated, indicating prolonged immune activation. MSCs exposed to isolated fracture serum demonstrated increased secretion of factors conducive to angiogenesis and isolated fracture healing (Saiz et al., 2025a). However, it is important to note that the *in vitro* component does not account for the secretions from other immune cells at the fracture site, particularly the well-documented cross-talk between MSCs and macrophages (Pajarinen et al., 2019). The *in vivo* studies revealed that systemically applied MSCs are less effective in modulating the local and systemic immune response, as they become entrapped in the lungs and do not migrate to the fracture site. Local application of MSCs in a hyaluronic acid hydrogel appeared to decrease systemic concentrations of pro-inflammatory cytokines, including IL-6, IL-17A, TNF-α, IFN-γ, IFN-β and IL-23, some of which interfere with bone remodeling pathways (Saiz et al., 2025a). The pro-inflammatory phenotype of endogenous MSCs in polytrauma could contribute to the dysfunction of MSC-mediated interactions at the fracture site. However, greater exploration and confirmation is required.

### 5.5 Other immune cells and trauma-associated myeloid cells

The impact of MSCs on other immune cells (CD4^+^ and CD8^+^ T cells, B cells, DCs and natural killer cells specifically) in polytrauma is not well studied. Although these cells are generally suppressed both locally and systemically in polytrauma (Saiz et al., 2025b), the mechanism underlying this suppression has only recently been elucidated. A study from Maniar et al. demonstrated that a CD11d+ trauma-associated immunosuppressive myeloid (TIM) cell appears to be an important contributor to the lack of activity in antigen-presenting cells and the adaptive immune system in a mouse polytrauma model (Maniar et al., 2025). Secretions from both M1-and M2-like macrophages such as IL-10 and TNF-α influence MSC effects in a variety of ways, so interaction with TIM cells may result in the aforementioned malfunction of MSC-mediated processes in polytrauma.

### 5.6 Complement-mediated MSC injury and lung sequestration

MSCs can act as both an effector and target in polytrauma, as demonstrated by the complement system. In concentrations consistent with polytrauma, both C3a and C5a enhanced MSC migratory activity, suggesting that complement activation at the injury site results in MSC recruitment (Huber-Lang et al., 2016). MSCs also have the capacity to both activate and inhibit the complement system: they generate C3 and C5 (which can be cleaved into C3a and C5a) and activate C3a-producing pathways, but also secrete factor H, an inhibitor of C3 cleavage and opsonization, demonstrating a self-regulatory mechanism (Huber-Lang et al., 2016). However, despite their expression of a variety of complement regulatory proteins (Cregs), they are frequently a target of opsonization in polytrauma: when compared to wild-type mice, C3-deficient mice experienced markedly decreased MSC injury (Huber-Lang et al., 2016; Li and Lin, 2012). This is likely a result of disruption of protective Cregs on the surface of MSCs, a common occurrence with other leukocytes early in polytrauma. Opsonization of MSCs could be a factor in why MSCs do not function properly in polytrauma. An overactive complement system could prevent MSCs from entering the bloodstream, impairing their ability to modulate the inflammatory response and migrate to the fracture site.

Another feasible explanation for the malfunction of MSC-mediated processes and the low blood concentration is that they become trapped in another part of the body. Several studies have demonstrated that intravenous infusion of MSCs results in accumulation of exogenous MSCs in the lungs. However, they are still able to exert some effects by secreting EVs and cytokines into the bloodstream (Saiz et al., 2025a; Eggenhofer et al., 2012; White et al., 2016). Other possible explanations are that an additional inhibitory influence acts on them (potentially the previously mentioned TIM cells) or that their effects are altered due to the overwhelming inflammatory response, similar to the pro-inflammatory phenotype noted in our previous work (Saiz et al., 2025a). Characterizing the mechanism by which MSC-mediated processes are impaired in polytrauma is critical to understanding how to treat polytraumatized patients better.

### 5.7 Challenges in modeling polytrauma

Although several studies have characterized the effects of polytrauma on both systemic and local immune cell activities, the literature on the direct impacts of MSCs on immune cell functions in polytrauma is limited. A big reason for this is the difficulty in simulating the effects of polytrauma on MSC activity, both *in vitro* and *in vivo*. Animal polytrauma models exist, but variables including patient genetics, age, comorbidities and the combination and severity of injuries impact the way that the immune system reacts to polytrauma (Hietbrink et al., 2006). Because polytrauma has a broad definition and the immune system can respond in various ways, identifying a combination of factors that accurately simulates the effects of polytrauma on MSCs is difficult. For example, Amann et al. used RNA sequencing, ELISA and multiplex assays of cell culture supernatant to compare the gene expression and protein secretion profile of MSCs stimulated with human polytrauma serum or inflammatory cues (Amann et al., 2019). They reported little overlap in differentially expressed genes between the group stimulated with polytrauma sera and both cocktail groups (Amann et al., 2019). Further *in vitro* studies to accurately simulate the effects of MSCs in polytrauma could elucidate the mechanisms by which MSC-mediated processes malfunction and support the use of MSCs as a therapeutic tool in polytrauma.

Overall, the evidence demonstrates a hostile immune environment that indicates dysfunction in several MSC-mediated functions in response to polytrauma, though the role and significance of MSCs in such an environment remains unclear. This emphasizes the need for greater characterization of the effects of native MSCs on immune cells in a polytrauma setting and an investigation into the use of MSCs as a tool in regulating the systemic inflammatory response. An *in vivo* polytrauma study investigating the actions of native MSCs and the decrease in blood concentration of MSCs is necessary to further understand the underlying mechanism of polytrauma. Additionally, the development of methods to accurately simulate the effects of MSCs in polytrauma *in vitro* is needed to allow for a simpler means of studying polytraumatized MSCs; isolating the effects from different injury combinations would be a good starting point. Tables 2, 3 provide a summary of the references used in the section, including the methods used, key findings and applications to the immunomodulatory capabilities of MSCs in polytrauma.

**TABLE 2:** Summary of key studies on MSCs in polytrauma and their immunomodulatory insights.

Study and model	Assays	Key finding	Limitations/translational interpretation	Ref
Human BM-MSC + neutrophil co-culture	Co-culture, ELISA, ROS, migration	MSCs inhibit neutrophil apoptosis via IL-6/STAT3 without inducing hyperactivation	In vitro only; non-trauma context; not validated in polytrauma serum	(Raffaghello et al., 2008)
Human MSC + serum; MSC in C3-deficient vs. WT mice	Flow cytometry, complement-deposition	MSCs activate complement and are targets of MAC; complement-deficient mice show improved MSC survival	Small in vivo model; mechanism shown, not clinical outcome	(Li and Lin, 2012)
Human MSC + Treg co-culture	Suppression assays, blocking antibodies	MSC-exposed Tregs gain suppressive capacity via IL-10/PD-1	In vitro; healthy-donor cells; effect in trauma milieu inferred	(Yan et al., 2014)
Murine immune-complex vasculitis + MSC	Histopathology, NET assays, SOD3 knockdown	MSCs engulf neutrophils (ICAM-1) and secrete SOD3 to prevent NETs/protease release	Non-fracture model; extrapolation to polytrauma indirect	(Jiang et al., 2016)
Human polytrauma patients (n = 11, ISS≥24) vs. controls	Flow cytometry, circulating MSC markers	Circulating MSC-marker + cells fall by 4 h; no mobilization surge	Small n; marker-based, not functional; supports supplementation	(Wiegner et al., 2018)
Human (n = 20, ISS≥16) + porcine polytrauma	Flow cytometry, Treg subsets	Treg proportion depressed through 72 h; partial recovery	Associative; does not prove MSC-driven Treg loss; validates porcine model	(Serve et al., 2018)
Human MSC + polytrauma sera vs. IL-1β vs. cocktails	RNA-seq, multiplex, ELISA	Trauma serum induces unique MSC gene programs; IL-1β only partially mimics	In vitro; underscores modelling difficulty; hypothesis-generating	(Amann et al., 2019)
Murine femur fracture + chest trauma	Flow, micro-CT, histology, SAXS	Sustained innate inflammation + suppressed adaptive immunity; impaired callus	Preclinical; defines dysregulation; MSC role inferred	(Saiz et al., 2025b)
Rat polytrauma scRNA-seq (blood, BM, callus)	scRNA-seq, flow, pathway analysis	CD11d+ TIM cells (systemic) and Spp1+ macrophages (local) drive suppression/fibrosis	Preclinical; candidate mediators, not proof	(Maniar et al., 2025)

Abbreviations**:** BM, bone marrow; WT, wild type; MAC, membrane attack complex; Treg, regulatory T cell; ISS, injury severity score; DEG, differentially expressed gene; PTCL, physiological trauma cocktail low; IHC, immunohistochemistry; SAXS, small-angle X-ray scattering; IVIS, *in vivo* imaging system; scRNA-seq, single-cell RNA sequencing; TIM, trauma-associated immunosuppressive myeloid cell; MSC, mesenchymal stromal cell; ELISA, enzyme-linked immunosorbent assay; qRT-PCR, quantitative reverse transcription polymerase chain reaction; ROS, reactive oxygen species; NET, neutrophil extracellular trap; SOD3, superoxide dismutase 3; ICAM-1, intercellular adhesion molecule 1; RNA-seq, RNA sequencing; micro-CT, micro-computed tomography; IV, intravenous; h, hour(s); d, day(s); wk, week(s); vs., versus; n, number; IL, interleukin; STAT, signal transducer and activator of transcription; PD-1, programmed cell death protein 1; TGF-β, transforming growth factor beta; CD, cluster of differentiation; FoxP3, forkhead box P3; C3, complement component 3; HA, hyaluronic acid-based hydrogel; P2, proline-rich intrinsically disordered peptide.

**TABLE 3:** Evidence-level framework for MSC-related mechanisms and applications in polytrauma.

Mechanism/application	Highest evidence tier	Direction of evidence	Key refs
MSC M1→M2 polarization impaired in polytrauma	Preclinical + in vitro; extrapolated	Plausible, not shown in humans	(Saiz et al., 2025b), (Ji et al., 2025)
Neutrophil hyperactivation/impaired MSC restraint	In vitro + non-trauma animal	Inferred; MSC role unproven	(Raffaghello et al., 2008), (Jiang et al., 2016), (Martí‐Chillón et al., 2023)
Treg depletion/reduced MSC-mediated support	Human + porcine (observational)	Associative; not causally MSC-driven	(Yan et al., 2014), (Serve et al., 2018)
Complement-mediated MSC injury	In vitro + animal	Demonstrated mechanistically; clinical impact unknown	(Huber-Lang et al., 2016), (Li and Lin, 2012)
Pro-inflammatory MSC secretome in polytrauma serum	In vitro + preclinical polytrauma	Direct (preclinical/*in vitro*)	(Saiz et al., 2025a), (Amann et al., 2019)
BMAC for nonunion	Human fracture/nonunion (non-polytrauma)	Established for isolated nonunion; extrapolated	(Desai et al., 2015), (Hernigou et al., 2006)
Culture-expanded MSC constructs for nonunion	Human Phase II (non-polytrauma)	Established for isolated nonunion; extrapolated	(Orozco Delclós et al., 2024)
Systemic MSC for trauma-related organ injury	Extrapolated (ARDS/sepsis) + preclinical	Safety shown; efficacy in trauma unproven	(Matthay et al., 2019), (He et al., 2018)
MSC-EVs in trauma/hemorrhagic shock	Preclinical only	Hypothesis-generating	(Valade et al., 2021)
Local scaffold-assisted MSC delivery in polytrauma	Preclinical polytrauma	Direct but animal-only	(Saiz et al., 2025a), (Saiz et al., 2025c)

## 6 Clinical and translational applications of MSCs in fracture healing and polytrauma

No controlled human trial has yet tested MSC or MSC-EV therapy in polytrauma for either fracture union or systemic immunomodulation. The evidence below is therefore largely indirect: it derives from human isolated fracture and nonunion studies, preclinical polytrauma models, *in vitro* MSC-stimulation studies, and extrapolation from MSC trials in inflammatory conditions such as ARDS and sepsis. Efficacy claims are framed accordingly.

### 6.1 MSC-based products for fracture healing and nonunion

Large bone defects and fracture nonunion remain challenging problems in orthopedics (Sheen et al., 2023). The current gold standard treatment for these orthopedic issues include surgical fixation with autologous bone grafting which can achieve very high union rates (∼97–99%) in atrophic nonunion (Desai et al., 2015). However, autografting has limitations including donor site morbidity (e.g., chronic pain), limited graft supply, and increased risk of infection (Desai et al., 2015). MSCs have therefore been explored as an alternative to enhance bone regeneration, and they can be obtained from various adult tissues (e.g., bone marrow, adipose tissue) as well as perinatal sources (Berebichez-Fridman and Montero-Olvera, 2018). These multipotent progenitor cells naturally home to injury sites (Rodríguez-Fuentes et al., 2021) and orchestrate repair by secreting anti-inflammatory, pro-angiogenic, and osteotropic factors while also differentiating into osteoblasts as needed (Garg et al., 2017). Thus, MSC-based therapies aim to harness the pro-healing capacity and osteogenic potential of these cells (Huber-Lang et al., 2016). This multifunctional regenerative role underlies clinical appeal of using MSCs for fracture healing.

BMSCs (bone marrow-derived stromal cells) are among the most extensively studied cell sources for skeletal tissue repair (Arthur and Gronthos, 2020). One widely studied approach involves percutaneous injection of MSC-rich bone marrow aspirate concentrate (BMAC) into the fracture site. Hernigou et al. demonstrated that concentrated autologous bone marrow grafting can effectively treat long bone nonunions (Hernigou et al., 2006). In a series of 60 atrophic tibial nonunions, an injection of ∼20 mL of concentrated bone marrow (averaging 50,000 colony-forming unit progenitors) achieved bony union in 53 patients (∼88% success) (Hernigou et al., 2006). Importantly, patients who healed had significantly higher concentrations of MSC progenitors in the graft compared to those that failed to heal (Hernigou et al., 2006). This study concluded that percutaneous autologous bone marrow MSC therapy is an effective and safe method for stimulating repair in nonunions if a sufficient number of progenitor cells is delivered (Hernigou et al., 2006). Subsequent clinical studies have built on this strategy by combining MSCs with osteoinductive or osteoconductive adjuvants. A retrospective cohort study at Hospital for Special Surgery treated 49 tibial delayed unions and nonunions with BMAC injections plus demineralized bone matrix (DBM) or recombinant BMP-2 (Desai et al., 2015). Radiographic union was achieved in the vast majority of cases, and notably there was no difference in healing rates between patients with small fracture gaps (<5 mm) and large gaps (>5 mm) (Desai et al., 2015). The MSC plus growth factor approach was effective even in challenging nonunions regardless of gap size or fracture location. The authors reported this combined treatment is safe and effective for delayed unions/nonunions with evidence that using DBM yielded higher union rates than BMP-2 in their cohort (Desai et al., 2015). This suggests MSC-rich bone marrow graft material can be potentiated by an appropriate scaffold or matrix creating an osteogenic environment conducive to healing. Importantly, these are isolated nonunion cohorts, not polytrauma populations.

More recently, tissue engineering strategies with culture expanded MSCs have entered clinical trials (Li et al., 2019). In a Phase II trial by Orozco Delclos et al., investigators implanted either allogeneic or autologous expanded adipose-derived MSCs on a tricalcium phosphate scaffold into atrophic long bone nonunions (Orozco Delclós et al., 2024). At 6 months follow up, 10 out of 12 treated nonunions (83%) achieved successful bone healing with MSC-seeded constructs (Orozco Delclós et al., 2024). The healing rate was comparable to a reference group treated with autologous bone marrow MSC grafts (79% union) indicating adipose-derived MSC “biografts” were highly effective. Importantly, the trial demonstrated that both autologous and allogeneic MSC sources were well tolerated and showed similar efficacy (Orozco Delclós et al., 2024). The ability to use allogeneic (donor-derived) MSCs without immune rejection suggests an off-the-shelf cell therapy could be feasible, sparing patients the difficulties of cell harvest and expansion. For instance, MSCs derived from perinatal tissues like Wharton’s jelly (umbilical cord) are highly proliferative and immunomodulatory, making them attractive allogeneic cell source that is being explored in other clinical contexts (Kamal and Kassem, 2020). The authors also reported no serious adverse events attributable to the MSC therapy, further confirming the safety of this approach in a clinical setting (Orozco Delclós et al., 2024). These findings align with broader systematic reviews indicating that MSC therapies have a strong safety profile across clinical trials with no major adverse events reported in applications ranging from orthopedics to cardiology (Rodríguez-Fuentes et al., 2021; Wang et al., 2018). Thus, in the context of fracture healing, MSC-based treatments, whether via BMAC or engineered cell/scaffold constructs, have shown promise in promoting union of complex fractures while maintaining an acceptable safety margin. Encouraging outcomes have been documented in early phase clinical studies, though the optimal cell dose, delivery method, and timing continue to be investigated (Perez et al., 2018). For instance, it remains unclear whether freshly isolated MSCs or *ex vivo* expanded cells yield superior healing. The impact of factors such as patient age or comorbidities (which can impair native MSC function) could also impact efficacy (Li et al., 2019; Perez et al., 2018). Nevertheless, for isolated fracture management, the translational trajectory is clear: MSC therapies are moving from bench to bedside in fracture management.

### 6.2 Systemic immunomodulation in trauma and critical illness

An important consideration is fracture healing may not occur in isolation, particularly in polytrauma patients who sustain multiple severe injuries. As previously stated, the systemic injury environment of polytrauma can profoundly influence bone repair, leading to poor healing outcomes (Champion et al., 1990). A robust initial inflammatory response is necessary for fracture healing, but severe trauma often triggers an exaggerated, dysregulated inflammatory cascade that may actually impair healing and contribute to nonunion (Loi et al., 2016; Maruyama et al., 2020; Huber-Lang et al., 2016). Huber-Lang et al. demonstrated the dual role of MSCs in trauma, describing MSCs as not just “actors” in healing (secreting cytokines, promoting angiogenesis, and differentiating into repair cells), but “targets” of the trauma milieu (Huber-Lang et al., 2016). Excessive inflammatory mediators, complement activation, and DAMPs released after major trauma can compromise endogenous MSCs, reducing their migratory and regenerative capacities and impairing fracture healing and tissue repair (Huber-Lang et al., 2016). Consequently, there is a significant translational interest in leveraging exogenous MSC therapy to counteract these effects not only to aid bone healing locally, but also to modulate the systemic inflammatory response after trauma. This overlap between fracture healing and polytrauma is where the dual regenerative and immunomodulatory functions of MSCs are especially valuable. Clinical scenarios such as a patient with multiple fractures, hemorrhagic shock, and SIRS illustrate the need for therapies that can address both bone regeneration and the prevention of organ failure (Huber‐Wagner et al., 2007).

As discussed above, polytrauma patients are at a high risk of developing systemic inflammatory response syndrome which can lead to subsequent complications such as multi-organ failure due to the massive release of inflammatory mediators (so-called “cytokine storm”) after trauma (Valade et al., 2021). In fact, severe trauma with hemorrhagic shock induces a complex pathophysiological response where tissue injury and hypoperfusion lead to activation of the innate immune system, complement, and coagulation pathways resulting in endothelial dysfunction, capillary leak, and widespread inflammation. If unmodulated, this hyperinflammatory state can lead to dysfunction and failure of a variety of organ systems, a major cause of late mortality in trauma patients (Valade et al., 2021). Moreover, prolonged inflammatory complications can lead to an immunosuppressive phase leaving patients vulnerable to sepsis. Given this complex immune dysregulation, polytrauma management has begun to look toward immunomodulatory therapies. MSCs have emerged as a compelling candidate in this context because of their potent ability to attenuate pathological inflammation and promote tissue repair simultaneously (Martí‐Chillón et al., 2023). Unlike traditional drugs that target single mediators, MSCs can act more broadly by interacting with multiple immune cell types and secreting trophic factors that help protect and regenerate injured tissue (Martí‐Chillón et al., 2023; Huber-Lang et al., 2016). Thus, MSCs could serve as a multimodal therapy in critical injury by tempering the harmful immune response that drives organ damage and potentially enhancing the repair of injured organs and bones. Martí‐Chillón et al. noted that MSCs have shown clinical benefits in certain inflammation-driven conditions (e.g., graft-versus-host disease and autoimmune disorders) and could be used for ICU patients with SIRS in the near future (Martí‐Chillón et al., 2023). Their review highlights the rationale that MSCs’ immunomodulatory, anti-inflammatory, and pro-regenerative properties could aid recovery in systemic inflammatory states like sepsis or trauma, although further clinical evidence is needed in the trauma/SIRS domain (Martí‐Chillón et al., 2023).

Early clinical trials have begun to evaluate MSC therapy in critical illness and trauma-related complications. These trials are in ARDS and sepsis, not polytrauma, and are cited here as indirect, extrapolated evidence. One of the main focuses has been ARDS, a severe lung condition that can occur after trauma or sepsis. In the phase 2a START trial, Matthay et al. tested a single intravenous dose of allogeneic bone marrow-derived MSCs in patients with moderate to severe ARDS (of various etiologies) (Matthay et al., 2019). The primary aim was to assess safety. The results showed that MSC infusion (10 million cells per kg) was well tolerated with no infusion-related hemodynamic or respiratory adverse events (Matthay et al., 2019). 28-day mortality did not differ significantly between the MSC-treated group and placebo in this small trial, but importantly there were no safety signals of concern (Matthay et al., 2019). The investigators noted that cell viability was variable and that larger trials (with possibly repeated dosing or higher quality cells) would be needed to determine efficacy (Matthay et al., 2019). Despite the lack of definitive efficacy in that study, the demonstration of safety in ARDS patients was an encouraging step. Similarly, in severe sepsis, a phase 1 dose escalation trial by He et al. evaluated umbilical cord derived MSCs given intravenously to 15 patients with septic shock (He et al., 2018). Patients received doses up to 3 × 10^6^ MSCs per kg. Consistent with the ARDS findings, no infusional toxicity or acute allergic reactions were observed, and there were no treatment-related serious adverse events (He et al., 2018). Although this phase 1 study was not designed to test efficacy, the treated septic patients showed a reduction in circulating inflammatory cytokine levels compared to matched controls suggesting a possible immunomodulatory effect (He et al., 2018). These early trials in sepsis and ARDS indicate the safety of administering MSCs to critically ill patients, laying the groundwork for larger efficacy trials. A comprehensive 2018 review of cell therapies in trauma corroborates this statement, concluding that MSC treatment appears safe in phase I/II settings but larger studies are needed to confirm clinical benefit (Wang et al., 2018). This is the current state of translation as safety has been established, and the field is now moving into phase II/III trials to rigorously test outcomes such as survival, organ function, and long-term recovery. No such trial has yet been conducted specifically in polytrauma.

### 6.3 Product definition, heterogeneity, and potency

MSC-based interventions are not a single product, and outcomes cannot be assumed to transfer between them. This review distinguishes MSC-rich bone marrow aspirate concentrate (BMAC), culture-expanded MSC products, MSC-derived extracellular vesicles (MSC-EVs), and biomaterial-assisted delivery of any of these. Even within culture-expanded products, therapeutic behavior is influenced by source tissue (bone marrow, adipose, umbilical cord/Wharton’s jelly), donor factors (age, comorbidity), passage number, priming/licensing status (e.g., IFN-γ, TNF-α exposure), cryopreservation and thaw, and the delivery matrix. Because of this lot-to-lot and donor-to-donor variability, robust release criteria and potency assays are essential.

No single assay captures MSC potency; a matrix approach is recommended (Christy et al., 2020; De Wolf et al., 2017; Chinnadurai et al., 2018). For polytrauma indication, potency readouts can be mapped directly to the mechanisms reviewed above: T cell suppression and regulatory T cell induction, macrophage M1→M2 polarization, neutrophil modulation, cytokine suppression (e.g., IDO/TSG-6 upregulation on inflammatory licensing), angiogenic support (endothelial tube-formation assays), and osteogenic support, with fracture union outcomes as the ultimate *in vivo* readout. Recent work has validated such assay matrices across multiple donors and lots, linking measured potency to secreted mediators including IDO1, TGF-β1, and CCL2 (Niekamp et al., 2026). Establishing which product attributes best predict a potent, pro-healing MSC is a prerequisite for consistent manufacturing.

### 6.4 Translational roadmap and barriers

Moving MSC-based approaches toward the polytrauma clinic requires confronting several barriers that are magnified relative to elective fracture care.

Timing of treatment can be a challenge. The biphasic SIRS/CARS response implies that a single dose is unlikely to be optimal. Staged strategies (early immunomodulatory dosing to temper hyperinflammation, later dosing to support regeneration) warrant preclinical testing. Delivery route and pulmonary entrapment can pose another issue. Intravenously infused MSCs are largely sequestered in the lungs and cleared rapidly, limiting delivery to the fracture site. Local, scaffold-assisted delivery avoids this and, in our polytrauma models, improved callus quality relative to systemic delivery. Procoagulant and thromboinflammatory risk presents another challenge which is particularly relevant in polytrauma. Culture-expanded MSCs express surface tissue factor (TF/CD142) and can trigger the instant blood-mediated inflammatory reaction (IBMIR), activating complement and coagulation upon blood contact. This effect is dose-, passage-, and source-dependent, being greatest for adipose-derived, intermediate for umbilical-cord, and lowest for bone marrow-derived MSCs (Moll et al., 2019; Christy et al., 2017; Moll et al., 2012). In a rat polytrauma/hemorrhage model, intravenous MSCs produced a dose-dependent coagulopathy and failed to correct acute traumatic coagulopathy (Wu et al., 2021), which is a critical caution given that polytrauma patients are frequently already coagulopathic. Proposed mitigations include assessing TF/CD142 expression and hemocompatibility as release criteria (Moll et al., 2022), preferring low passage or bone marrow-derived cells, favoring local delivery, and co-administering heparin. Immunogenicity and repeat dosing represent additional barriers. Allogeneic “off-the-shelf” products are attractive for acute trauma but raise questions of sensitization with repeat dosing and of donor-dependent efficacy that require dedicated study. Manufacturing, release criteria, and regulatory pathway are other considerations. Consistent manufacturing, defined potency assays, and clear release specifications are prerequisites for regulatory approval; cell products and cell-free EV products follow distinct regulatory pathways. Lastly, patient heterogeneity is a unique barrier. The variability of injury patterns, comorbidities, and immune trajectories in polytrauma argues for stratified or personalized approaches rather than a uniform protocol.

### 6.5 MSC- derived extracellular vesicles

Beyond whole cell therapy, researchers are also exploring MSC derived biologics as a novel strategy in trauma care. EVs secreted by MSCs are under investigation as a cell-free therapeutic product. MSC-derived EVs (which include exosomes) carry many of the cytokines, growth factors, and microRNA signals of the parent cells and can exert similar immunomodulatory effects without the logistical complexities of live cell administration (Valade et al., 2021). MSC-EV preparations are, however, heterogeneous, and rigorous work should conform to the International Society for Extracellular vesicles MISEV2023 framework for EV nomenclature, separation from non-vesicular particles, and characterization (Welsh et al., 2024). Outstanding hurdles for cell-free products include scalable and reproducible manufacturing, definition of potency assays, standardization of dosing and targeting, and a regulatory pathway distinct from that of cell products. Importantly, all MSC-EV data in the trauma/hemorrhagic-shock setting remain preclinical, and procoagulant surface molecules (tissue factor, phosphatidylserine) can also be carried by EVs, so hemocompatibility applies to cell-free products as well. This could be especially advantageous in acute trauma settings as EVs can be stored, standardized and given immediately, whereas cell therapies often require preparation time. A review from Valade et al. described how the therapeutic potential of MSC-EVs can attenuate the detrimental immune response after trauma (Valade et al., 2021). Although the review evaluated animal models with hemorrhagic shock, administration of MSC-EVs has been shown to reduce lung injury and improve neurologic outcomes by modulating inflammatory pathways (Valade et al., 2021). These findings imply that the benefits of MSCs in trauma (e.g., reducing capillary leak, cytokine storm and promoting tissue repair) might be recapitulated by MSC vesicles. As a result, there is enthusiasm for developing cell-free MSC therapies that could be rapidly deployed in the field or emergency department for trauma patients (Valade et al., 2021). Such EV-based approaches remain in preclinical or early investigational stages but represent an important translational Frontier derived from MSC research.

Overall, the clinical translation of MSCs in trauma spans both local applications and systemic therapies. In fracture healing, MSC-based interventions (bone marrow concentrates, MSC-seeded scaffolds, etc.) have shown success in promoting union of complex fractures and nonunion offering an adjunct to or replacement for traditional bone grafts (Desai et al., 2015; Orozco Delclós et al., 2024). The therapeutic and delivery studies discussed in this section are summarized in Table 1. These therapies leverage the osteogenic and pro-regenerative capacity of MSCs to enhance skeletal repair. In the polytrauma and critical care realm, MSC infusion is being investigated to treat the systemic consequences of trauma, namely, excessive inflammation and organ damage. To date, however, this evidence is drawn from ARDS and sepsis rather than polytrauma. Early clinical studies in ARDS and sepsis suggest that MSC therapy is safe in severely ill patients, and laboratory evidence points to meaningful immunomodulatory effects that could translate into improved outcomes (Matthay et al., 2019; He et al., 2018). Looking ahead, ongoing trials are testing whether MSCs can reduce complications like ARDS, AKI, or MODS/MOF in trauma patients, and whether such interventions ultimately improve survival or functional recovery. Furthermore, the overlap between fracture healing and polytrauma management is a notable area of interest, as polytraumatized patients with orthopedic injuries might benefit from MSCs both to accelerate bone healing and mitigate systemic SIRS. The ability of MSCs to act on both fronts in bone regeneration and immune regulation is unique and underlies their appeal in trauma medicine (Martí‐Chillón et al., 2023; Huber-Lang et al., 2016). While challenges remain (such as optimizing cell dose, timing, and ensuring transplanted cell viability), investigators are exploring priming (preconditioning) strategies to further enhance MSCs therapeutic potency. The current state of the literature suggests that MSC-based therapies are advancing along the translational pathway, although, as noted above, no controlled human trial has yet tested MSC or MSC-EV therapy specifically in polytrauma. In the coming years, well-designed clinical trials will clarify the true efficacy of MSCs in fracture healing and polytrauma, potentially establishing new regenerative treatment paradigms in both areas.

## 7 Future perspectives

### 7.1 Mechanistic uncertainties and paradoxes

Despite significant progress in elucidating the function of MSCs in fracture healing, fundamental mechanistic uncertainties and paradoxes persist. For instance, MSC-driven immunomodulation appears highly context-dependent, with certain inflammatory cues yielding conflicting outcomes on bone repair. Pro-inflammatory signals are essential for initiating regeneration, yet excessive or prolonged inflammation can derail healing (Loi et al., 2016; Maruyama et al., 2020). Some studies report that inflammatory cytokines impede osteogenic differentiation of MSCs, whereas others find that they can enhance bone formation under specific conditions, suggesting that factors such as cytokine dose, timing, or species differences may explain the discrepancies. Likewise, MSCs have demonstrated seemingly paradoxical behaviors in immune regulation: they can secrete factors that dampen neutrophil recruitment (e.g., TSG-6) but also produce chemokines like IL-8 that increase neutrophil migration in certain contexts (Xu et al., 2023). The contradictions underscore the need for future research to determine the conditions under which MSCs exert beneficial versus detrimental effects. Clarifying how variables such as the local cytokines, hypoxia, or cellular crosstalk dictate MSC behavior will be crucial to resolving these inconsistencies. In particular, whether key immunomodulatory processes such as the MSC-induced shift of macrophages from an M1-like to M2-like phenotype are truly impaired in polytrauma patients remains an open question (Saiz et al., 2025b). Addressing these knowledge gaps will inform more precise therapeutic targeting, ensuring that pro-regenerative MSC functions can be harnessed while their potentially harmful or counterproductive actions are avoided.

### 7.2 Representative polytrauma models

A major challenge moving forward is capturing the complex, multifactorial environment of polytrauma in experimental models. Polytrauma creates a storm of systemic perturbations from hemorrhagic shock and massive release of DAMPs to surges of catecholamines and complement activation that converge to influence MSC function in ways we are only beginning to understand. Intrinsic patient factors (e.g., age, genetics, comorbidities) and extrinsic injury factors (combination and severity of injuries) further diversify the trauma response, making it difficult to reproducibly simulate *in vitro* or in preclinical animal studies (Hietbrink et al., 2006). Notably, even sophisticated *in vitro* approaches have struggled to replicate the polytrauma milieu (Amann et al., 2019). This further suggests that the combined effect of multiple injuries exceeds the sum of their individual parts, and unrecognized or synergistic factors in polytrauma sera may be crucial for accurately modeling MSC behavior (Amann et al., 2019). Future studies should prioritize developing more representative experimental models. For example, multifactorial bioreactor systems or advanced trauma simulation animal models can faithfully recreate the polytrauma immune environment. Such models may help pinpoint how and why endogenous MSC-mediated processes fail under extreme systemic stress, and how we might intervene to restore their function. In parallel, comparative clinical studies in polytrauma versus isolated fracture patients (leveraging biobanked serum, cells, and metadata) could illuminate which immunological and metabolic disturbances are most responsible for derailing MSCs *in vivo*. Additionally, isolating the different immune responses to various injury combinations in preclinical animal models (fracture + traumatic brain injury, fracture + chest trauma, etc.) would allow for a more nuanced approach to the study of endogenous MSCs in polytrauma.

### 7.3 Understudied cellular interactions

Another avenue for future investigation is the array of under-studied cellular interactions and pathways involving MSCs during trauma recovery. While MSC crosstalk with macrophages is well documented, their influence on other immune populations in trauma remains poorly characterized. For instance, the impact of MSCs on T cell, B cell, or NK cell activity in polytrauma has received little attention in the literature. Severe trauma can induce a state of broad immune suppression affecting these cell types (Hoff et al., 2017), and recent work identified a novel CD11d^+^ TIM cell that may contribute to this immune paralysis (Maniar et al., 2025). How such immunosuppressive myeloid cells and trauma-altered regulatory T cell dynamics intersect with MSC function is also an open question (Maniar et al., 2025; Serve et al., 2018). Discovering these relationships is important for designing MSC-based interventions that account for the broader immune context. Both a rigorous mechanistic study and the development of an accurate way to simulate the effects of polytrauma on MSCs *in vitro* are needed to characterize the effects of MSCs in polytrauma and allow for further research in the field. Similarly, the role of the complement system at the injury site merits deeper exploration. Complement fragments like C3a/C5a can recruit MSCs to injury sites, but uncontrolled complement activation can also opsonize and injure MSCs. Going forward, studies should investigate strategies to protect MSCs from complement-mediated damage. Enhancing expression of complement regulatory proteins or using complement inhibitors without impeding positive migratory signals could prove beneficial (Huber-Lang et al., 2016; Li and Lin, 2012). Finally, the intrinsic heterogeneity of MSC populations represents another underappreciated variable. MSCs from different tissue sources or donors, or even subpopulations within a given sample can have divergent differentiation and immunomodulatory capacities (Wang et al., 2021). Future work employing single cell analyses and functional assays should aim to identify the most therapeutically potent MSC subsets and determine how trauma-related factors might differentially affect these subsets. By mapping the phenotypes of “responder” vs. “non-responder” MSCs under inflammatory stress, cell-based therapies can advance with more consistent efficacy.

### 7.4 Delivery, priming, and timing

On the translational front, the promise of MSC-based therapies for challenging fracture healing scenarios has grown, but significant practical challenges remain before these approaches can fully deliver on their potential in polytrauma patients. One barrier is effective delivery and engraftment of MSCs in the hostile post-injury milieu. Systemically administered MSCs tend to sequester in the lungs after intravenous infusion, with minimal homing to other organs or injured tissues (Eggenhofer et al., 2012; White et al., 2016). This pulmonary trapping phenomenon, while potentially beneficial for treating acute lung injury, limits the cells’ availability to migrate to fracture sites in a polytrauma scenario. Even the body’s own circulating MSCs are found at reduced levels in severely injured patients, and it is unclear whether sufficient numbers reach the bone injury to exert local effects (Wiegner et al., 2018). To overcome this, future therapies may need to optimize cell delivery methods: for example, using intravascular delivery routes that bypass the lung first pass effect (such as intra-arterial or intraosseous injections to target the fracture zone), or encapsulating MSCs in biomaterial carriers that can be implanted directly at injury sites. Transplanting MSCs as cellular aggregates (spheroids) can prolong their survival and increase endogenous secretion of bioactive factors (Griffin et al., 2022). Combining MSCs with supportive scaffolds or matrices is a promising strategy already yielding positive results in early studies. By providing a hospitable niche, scaffolds may improve MSC retention, viability, and localized action in an inflammatory wound bed. In parallel, priming or preconditioning MSCs before delivery is an active area of research aimed at boosting their resilience and functional potency (Ho et al., 2018; Beegle et al., 2015). Various priming approaches such as exposure to hypoxia, inflammatory cytokines, growth factors, or pharmacological agents have been shown to enhance specific therapeutic properties of MSCs (e.g., secretion of anti-inflammatory factors or survival in oxidative stress conditions). For example, IFN-γ or TNF-α can enhance MSC immunosuppressive capacity, whereas hypoxic preconditioning may improve their pro-angiogenic and migratory abilities. Tailoring these priming strategies to address the polytrauma environment (for instance, upregulating anti-apoptotic and antioxidant defenses in MSCs prior to infusion) could significantly improve their performance *in vivo*. Early investigations indicate that primed MSCs exhibit distinct and enhanced therapeutic capabilities compared with unmodified MSCs (Miceli et al., 2023). Refining the timing of administration will also be critical: it is conceivable that staged MSC therapy (e.g., an initial dose to moderate the hyperinflammatory phase of trauma, followed by a later dose to promote regeneration) may yield better outcomes than a single intervention. Each of these variables such as delivery route, scaffold context, cell priming, and timing should be systematically explored in preclinical models to identify optimal protocols for clinical testing (Li et al., 2019; Perez et al., 2018). Encouragingly, the safety profile of MSC therapies remains robust across numerous trials, and dosing/toxicity limits have not been a major concern, which affords some flexibility to experiment with these parameters (Rodríguez-Fuentes et al., 2021).

### 7.5 Extracellular vesicles and off-the-shelf products

The therapeutic outlook for MSCs in fracture healing and immune modulation is further bolstered by emerging concepts that go beyond direct cell transplantation. One such concept is the use of MSC-derived products, particularly EVs and exosomes as surrogates for MSC therapy. MSC-derived EVs carry many of the cytokines, growth factors, and miRNAs that mediate immunomodulation and tissue repair. Notably, preclinical work has shown that MSC exosomes can enhance bone regeneration and angiogenesis in nonunion models and even protect organs from inflammatory injury in trauma-like conditions (Valade et al., 2021). The effective development and use of EVs may enable clinicians to circumvent challenges of cell survival, immune rejection, or lung entrapment, while still capitalizing on MSCs’ paracrine healing powers. Future research should investigate EV-based therapies in the context of polytrauma, including optimal isolation methods, dosing, and targeting strategies to ensure these vesicles reach damaged tissues. Another promising direction is the pursuit of “off-the-shelf” allogeneic MSC therapies. Recent work from our group has demonstrated a significant enhancement in murine polytrauma fracture healing through local delivery of MSCs and MSCs with P2, a proline-rich intrinsically disordered peptide (Saiz et al., 2025a; Saiz et al., 2025c). Local delivery involved injection of these factors via hyaluronic acid-based hydrogel. This basic science research paves the way for further investigation into the use of MSCs and intrinsically disordered peptides in clinical settings. Further, recent clinical studies have provided proof of concept that allogeneic MSCs can be used safely with therapeutic benefit comparable to autologous cells. In a phase II trial for nonunion fractures, donor-derived MSCs on a scaffold achieved union rates similar to autologous MSC grafts with no immune-related complications (Orozco Delclós et al., 2024). This opens the door for readily available MSC treatments in acute settings like trauma, where waiting to harvest a patient’s own cells may not be feasible. However, allogeneic use also raises questions that warrant study: How do donor cell characteristics (health, age, tissue source) influence efficacy? Could repeated dosing with allogeneic MSCs induce subtle immune sensitization over time? And importantly, how does MSC heterogeneity impact outcomes when cells are sourced from different donors or expanded in culture? The finding that intrinsic cell heterogeneity can affect MSC behavior more than storage conditions highlights the importance of developing robust release criteria and perhaps potency assays for MSC products (Wang et al., 2021). As MSC therapeutics move towards standardization, the field must establish the cell properties (surface markers, secretome profile, metabolic status, etc.) that best predict a potent pro-healing MSC and ensure consistent manufacturing to those specifications.

In summary, a holistic, systems-level approach will be essential for translating MSC research into meaningful clinical advances, especially in the complex scenario of polytrauma. Unlike isolated fractures, polytrauma involves a convergence of systemic inflammation, immune dysfunction, and multi-organ stress that demands therapeutic strategies capable of multi-target regulation (Figure 4). MSCs are uniquely appealing in this regard because of their dual capacity to orchestrate repair and modulate immune responses. Yet, leveraging this capacity in practice will require integrating insights across disciplines. Future studies should bridge the gap between osteogenesis and immunology in trauma; for example, understanding how the metabolic and endocrine upheaval (hypercatecholaminemia, insulin resistance, acute phase responses) affect MSC signaling and bone regeneration. There is also a need for carefully designed clinical trials in trauma populations to test MSC interventions at the bedside. Early-phase trials in analogous inflammatory conditions, such as sepsis and acute respiratory distress syndrome, have demonstrated that MSC administration is feasible and safe in critically ill patients, laying important groundwork (Matthay et al., 2019; He et al., 2018). Building on these findings, trauma-specific trials can evaluate whether timely MSC or MSC-EV therapies reduce complications like SIRS, prevent secondary organ failure, or expedite fracture union in polytrauma patients. Ultimately, the future direction of this field is moving toward combination and personalized therapies: MSC-based treatments may be most effective when combined with supportive modalities (e.g., anti-inflammatory drugs, bioactive scaffolds, or growth factors) and tailored to the individual patient’s injury profile and immune status. By addressing the current mechanistic gaps and translational hurdles such as deciphering MSC’s context specific behavior to refining delivery and enhancing their functions, we can better realize the full therapeutic promise of MSCs. In doing so, it may become possible to modulate the post-trauma immune environment in a way that both mitigates systemic injury and promotes robust healing, fundamentally improving outcomes for patients suffering severe injuries.

**FIGURE 4:**
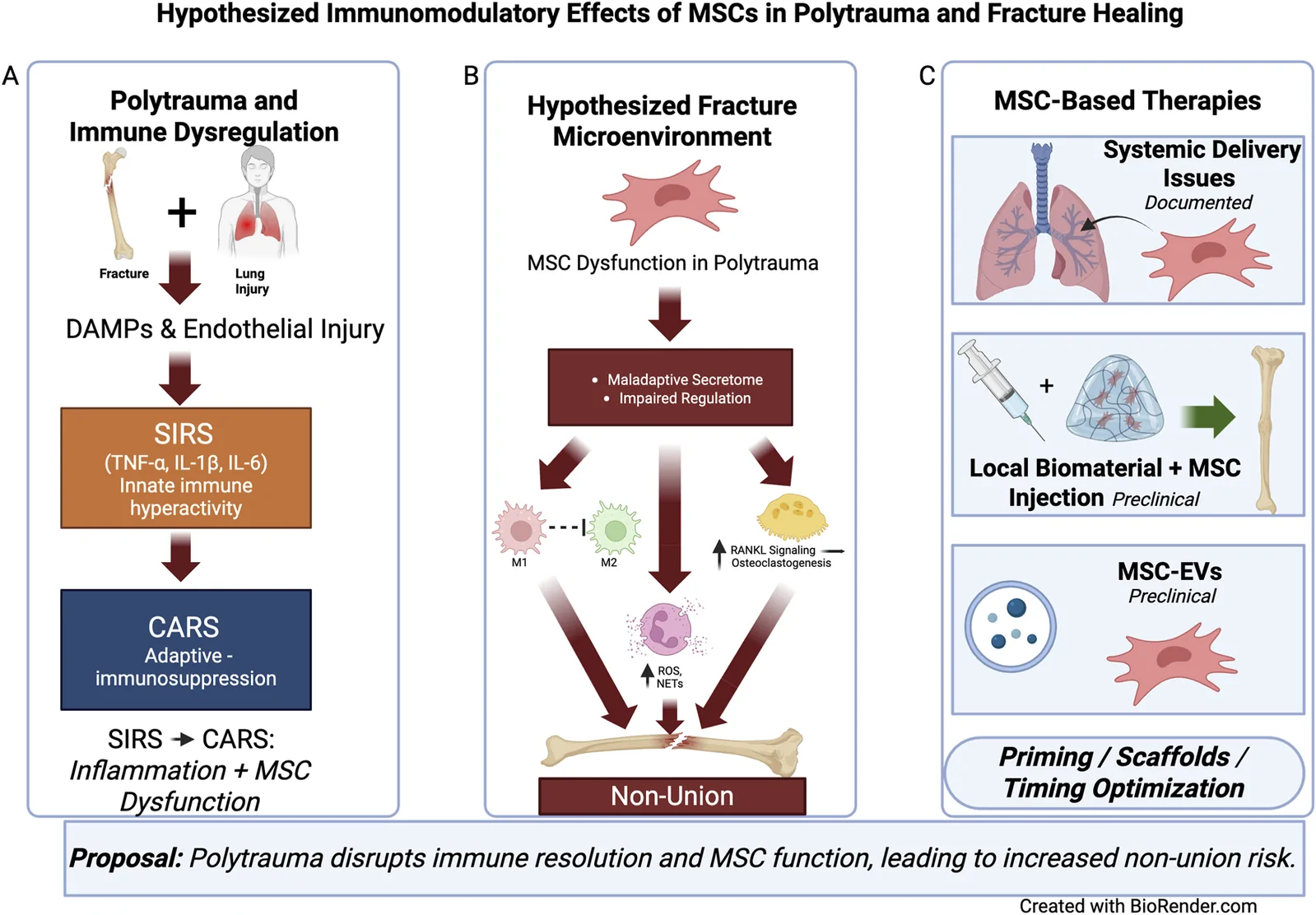
Overview of the Immunoregulatory Role and Therapeutic Potential of MSCs in Polytrauma. **(A)** Polytrauma induces tissue and endothelial injury with release of damage associated molecular patterns (DAMPs), driving an early hyperinflammatory systemic inflammatory syndrome (SIRS) followed by a compensatory anti-inflammatory response syndrome (CARS). This maladaptive immune transition may disrupt immune resolution and impair MSC function. **(B)** Within the fracture microenvironment, hypothesized MSC dysfunction leads to a maladaptive secretome, impaired macrophage polarization, persistent neutrophil activation, and increased RANKL-mediated osteoclastogenesis, which may collectively contribute to impaired fracture healing and nonunion. **(C)** MSC-based therapeutic strategies include systemic delivery, local biomaterial-based MSC delivery, and MSC-derived extracellular vesicles (MSC-EVs). Optimization of delivery route, priming, scaffolds, and timing may restore immune bone coupling and improve fracture healing following polytrauma. Created using BioRender.

## 8 Conclusion

MSC immunomodulation coordinates the transition from inflammation to regeneration in fracture healing, promoting angiogenesis and osteogenesis while affecting macrophage polarization, neutrophil activity, and regulatory T cell responses. Polytrauma imposes a systemic imbalance associated with prolonged inflammation, immune suppression, and higher nonunion risk, in which several MSC mediated functions appear blunted. However, direct evidence that endogenous MSCs are dysfunctional in human polytrauma remains limited, and no controlled human trial has tested MSC or MSC-EV therapy in this setting. The priority gaps are therefore: (1) representative polytrauma models that reproduce the systemic milieu; (2) product definition and validated, mechanism-linked potency assays; and (3) hemocompatibility-aware delivery strategies. Addressing these gaps will determine whether MSC-based, EV-based, or scaffold-assisted approaches can safely couple systemic immunomodulation with local bone repair in polytrauma patients.

## Glossary

M1: macrophage type 1
M2: macrophage type 2
MSC: mesenchymal stromal cell (also termed mesenchymal stem/stromal cell; “mesenchymal stromal cell” used as the preferred term throughout)
TGF-β: transforming growth factor-β
BMP-2,7, etc.,: bone morphogenetic proteins
IL-1, 4, 10, etc.,: interleukin
IFN-γ: interferon-gamma
TNF-α: tumor necrosis factor alpha
OSM: oncostatin M
RANKL: Receptor Activator of Nuclear Factor-kappa B Ligand
NF-κB: nuclear factor kappa-light-chain- enhancer of activated B cells
BMSCs: bone marrow stromal/stem cells
ROS: reactive oxygen species
PAMPs: pathogen-associated molecular patterns
DAMPs: damage-associated molecular patterns
ARDS: acute respiratory distress syndrome
AKI: acute kidney injury
VEGF: vascular endothelial growth factor
OPG: osteoprotegerin
PGE2: prostaglandin E2
BM-MSC: bone marrow-derived MSC
EV: extracellular vesicles
IDO: indoleamine 2,3 dioxygenase
HO-1: heme oxygenase-1
SDF-1α: stromal cell-derived factor 1 alpha
ISS: injury severity score
MODS: multi-organ dysfunction syndrome
MOF: multi-organ failure
CARS: compensatory anti-inflammatory response
TLRs: toll like receptors
PRRs: pattern recognition receptors
CCL2: chemokine (C-C motif) ligand two
CXCL8: chemokine (CXC motif) ligand eight
STAT1,3,6, etc.,: signal transducer and activator of transcription
IRF5: interferon regulatory factor 5
Th1: T helper one
Th2: T helper two
RORγt: retinoid-related orphan receptor gamma t
FOXP3: forkhead box P3
Tregs: Regulatory T cells
IGF-1: insulin-like growth factor 1
**CD4** ^**+**^: cluster of differentiation four positive T cell
**CD8** ^**+**^: cluster of differentiation eight positive T cell
CXCL12: C-X-C motif chemokine ligand 12
TNFR1/2: tumor necrosis factor receptor 1/2
PI3K/Akt: phosphatidylinositol 3-kinase/protein kinase B (Akt)
JAK/STAT: janus kinase/signal transducer and activator of transcription
miR-21: microRNA-21
miR-146a: microRNA-146a
*RUNX2*: runt-related transcription factor 2
SOX9: SRY-box transcription factor 9
EP2: prostaglandin E2 receptor subtype 2
EP4: prostaglandin E2 receptor subtype 4
M0: macrophage type 0
NET: neutrophil extracellular trap
SOD3: superoxide dismutase
PD-1: programmed cell death protein one
CTLA-4: cytotoxic T lymphocyte antigen-4
LAG-3: lymphocyte-activation gene three
CD11d: αD subunit of the αDβ2 integrin
TIM: trauma associated immunosuppressive myeloid
C3, C3a, C5a: complement 3, 3a, 5a
RNA: ribonucleic acid
ELISA: enzyme linked immunosorbent assay
*SPP1*: Secreted Phosphoprotein one (osteopontin)
BMAC: bone marrow aspirate concentrate
MSC-EV: mesenchymal stromal cell-derived extracellular vesicle
TF: tissue factor
IBMIR: instant blood mediated inflammatory reaction
MISEV2023: Minimal Information for Studies of Extracellular Vesicles 2023
